# Changes in DNA 5-Hydroxymethylcytosine Levels and the Underlying Mechanism in Non-functioning Pituitary Adenomas

**DOI:** 10.3389/fendo.2020.00361

**Published:** 2020-07-08

**Authors:** Yiwen Xu, Yamei Niu, Kan Deng, Hui Pan, Feng Feng, Fengying Gong, Wei-Min Tong, Shi Chen, Lin Lu, Renzhi Wang, Hui You, Yong Yao, Huijuan Zhu

**Affiliations:** ^1^Department of Pediatrics, Peking Union Medical College Hospital (PUMCH), Chinese Academy of Medical Sciences and Peking Union Medical College (CAMS & PUMC), Beijing, China; ^2^Department of Pathology, Institute of Basic Medical Sciences Chinese Academy of Medical Sciences and Peking Union Medical College; Neuroscience Center, Chinese Academy of Medical Sciences, Beijing, China; ^3^Department of Neurosurgery, Peking Union Medical College Hospital (PUMCH), Chinese Academy of Medical Sciences and Peking Union Medical College (CAMS & PUMC), Beijing, China; ^4^Key Laboratory of Endocrinology of National Health and Family Planning Commission, Department of Endocrinology, Peking Union Medical College Hospital (PUMCH), Chinese Academy of Medical Sciences and Peking Union Medical College (CAMS & PUMC), Beijing, China; ^5^Department of Radiology, Peking Union Medical College Hospital (PUMCH), Chinese Academy of Medical Sciences and Peking Union Medical College (CAMS & PUMC), Beijing, China

**Keywords:** 5hmC, NFPAs, TET2, ultrahigh performance liquid chromatography-electrospray ionization-tandem mass spectrometry (UPLC-ESI-MS/MS), DNA demethylation

## Abstract

Epigenetic factors have been proven to contribute to pituitary adenoma formation. 5-hydroxymethylcytosine (5hmC), which is catalyzed by ten-eleven translocation 2 (TET2), is related to DNA demethylation. In order to explore the pathogenesis of non-functioning pituitary adenomas (NFPAs), we detected genomic 5hmC levels in 57 NFPAs and 5 normal pituitary glands, and TET2 expression, distribution and *TET2* alteration. Genomic 5hmC levels in NFPAs were significantly lower than those in normal pituitary glands (0.38‰ (0.24‰, 0.61‰) vs. 2.47‰ (1.56‰, 2.83‰), *P* < 0.0001). There was positive correlation of 5hmC levels with TET2 total and nuclear expression in NFPAs (*r* = 0.461, *P* = 0.018; *r* = 0.458, *P* = 0.019). Genomic 5hmC levels in NFPAs with TET2 p.P29R were significantly lower than those in wild type NFPAs (0.33 ± 0.18‰ vs. 0.51 ± 0.25‰, *P* = 0.021). We found genomic 5hmC loss in human NFPAs for the first time. Genomic 5hmC levels may be affected by TET2 expression, subcellular localization and *TET2* mutation.

## Background

Pituitary adenomas (PAs) derive from adenohypophysis and account for 15–25% of all intracranial tumors (1, 2). PAs induce severe clinical symptoms and the treatment remains intractable. The pathogenesis of PAs are associated with activation of oncogenes, inactivation of tumor suppressor genes, changes in epigenetic modification, dysregulation of miRNAs, dysregulation of cytokines and growth factors, hormone stimulation, etc. Despite of these findings, the exact mechanism is still unclear (1, 3). With the development of epigenetic studies, many researchers think that changes in epigenetic modification may play an important role in PA formation. Epigenetic changes have been revealed in tumor suppressor genes, such as *CDKN1A, GADD45y, FGFR2*, and *caspase-8*; oncogenes, such as *MAGEA3*, and *PTTG;* imprinted genes, such as *GNAS1, NNAT*, and *MEG3*; epigenome modifiers, such as *DNMT3b*; and transcription regulators, such as *Ik, HMGA2* (4).

5-hydroxymethylcytosine (5hmC), hydroxymethylation of cytosine of DNA, is a type of epigenetic modification. Bhattacharyya *et al*. reported that increased 5hmC in the promoter of *GATA6* resulted in GATA6 overexpression (5). 5-methylcytosine (5mC), methylation of cytosine, is catalyzed by ten-eleven translocation (TET) into 5hmC, 5fC and 5caC stepwise. Kober *et al*. found that with higher promoter DNA methylation, the expression of *SFN, STAT5A* and *DUSP1* decreased (6). 5hmC, 5fC, and 5caC can go back to cytosine by base excision repair. Thus, 5hmC is a key intermediate molecule in DNA demethylation and is closely related to gene expression regulation. Many studies showed great differences of 5hmC in tumors and normal tissue (7). Genomic 5hmC levels decreased in malignant tumors (8) but increased in benign uterine leiomyoma (9). In addition, 5hmC is concentrated in the promoter region of the oncogene *GATA6* in pancreatic cancer. This phenomenon occurs along with changes in *GATA6* methylation and transcription (5). Therefore, changes in expression of tumor-related genes mediated by 5hmC may related with tumor formation. Moreover, genomic 5hmC levels are related with the invasion and prognosis of some tumors. For example, genomic 5hmC levels were negatively related to invasive melanoma depth (10). Reduction of 5hmC was a marker for poor prognosis in estrogen receptor/progesterone receptor (ER/PR)-negative patients with breast cancer (11).

Genomic 5hmC levels are affected by the expression and function of TET. The function of TET is affected by gene mutations and protein localization. The TET family includes TET1, TET2 and TET3, and they have a similar construction and function. Their catalytic domain is located in the C-terminus, and the DNA recognition domain is located in N-terminus. However, TET2 does not have a DNA recognition domain and requires association of a helper, named inhibition of the Dvl and Axin complex (IDAX) (12, 13). In the TET family, most mutations occur in *TET2*. There are more than 200 mutations in *TET2* in myeloid disease. p.H1802Q, p.H1802R and p.R1817S in TET2 induced the reduction of genomic 5hmC levels (14, 15). Additionally, TET2 cytoplasmic localization occurs in colon cancer but not in normal tissue. Reduction of TET2 nuclear localization may be responsible for the decrease in genomic 5hmC, and TET2 cytoplasmic localization was related with invasive colon cancer subtype (16). Therefore, we explored TET2 in our study. TET1 and TET3 may be explored in future studies.

PAs do great harm to human health, so it is important to uncover mechanism of PAs. Epigenetic modifications may be responsible for the formation of PAs. There is no study regarding genomic 5hmC levels in PAs, so our study aimed to explore genomic 5hmC levels in PAs and the underlying mechanism. We hope to add new knowledge to the epigenetic mechanism involved in PAs. It is unclear whether the pathogenesis of different subtypes of PAs are the same. We are also unsure whether hormone secreting interferes with results, so we choose non-functioning pituitary adenomas (NFPAs), a PA subtype without obvious hormone secretion, to prevent confusion of other PA subtypes.

## Materials and Methods

### Samples and NFPA Invasion Criteria

Samples of NFPAs were obtained from patients in Peking Union Medical College Hospital (PUMCH) from December 2013 to November 2015. Patients were included according to the following criteria: samples were pathologically diagnosed of PAs; there was neither increased anterior pituitary hormones in the blood nor clinical manifestations due to increased anterior pituitary hormones. Normal pituitary glands, which were donated, were obtained from the Chinese Academy of Medical Sciences (CAMS) Brain Bank. Normal pituitary glands were obtained from corpses without cerebral diseases. There were 57 NFPAs and 6 normal pituitary glands (a sample was only used for immunohistochemistry). We separated NFPAs into invasive and non-invasive groups according to the MRI results and surgery records. This work was, respectively, conducted by two experienced neurosurgeons in PUMCH.

### DNA Extraction and Ultrahigh Performance Liquid Chromatography-Electrospray Ionization-Tandem Mass Spectrometry (UPLC-ESI-MS/MS) Preparation

A DNA extraction kit (TianGen, DP304-03, Beijing, China) was used to isolate genomic DNA following the protocol in the instructions of the DNA extraction kit. Samples (NFPAs and normal pituitary glands) were homogenized using a tissue grinder. Two hundred microliter of GA and 4 μl of RNase A were added. Samples were mixed by vortex and incubated for 5 mins. Twenty microliter of proteinase K was added. Samples were incubated at 56°C at 500 rpm for 3 h or overnight prior to the addition of 200 μl of GB. Samples were incubated at 70°C for 10 mins, and then the supernatant was separated. Two hundred microliter of 100% ethanol was added to the supernatant. Then, the supernatant was transferred to spin columns and centrifuged at 13,300 rpm for 1 min. Five hundred microliter of GD was added into the spin columns and then centrifuged at 13,300 rpm for 1 min. Six hundred microliter of PW was added to the spin columns and then centrifuged at 13,300 rpm for 1 min. Another 600 μl of PW was added to the spin columns and then centrifuged at 13,300 rpm for 1 min again. The liquid was discarded and 50 μl of ddH_2_O was added. Five mins later, the samples were centrifuged at 13,300 rpm for 2 mins. Genomic DNA was present in the liquid.

1.5 μg of genomic DNA was added to 40 μl of ddH_2_O. After incubation at 100°C for 3 mins, the mixture was put on ice immediately. Four microliter of 0.1 M NH_4_COOH (PH 5.3) and 3 μl of 1 U/μl nuclease P1 were added. After incubation at 42°C for 6 hours, 4.7 μl of 1 M NH_4_HCO_3_ and 1.5 μl of 1 U/μl alkaline phosphatase were added. After incubation at 37°C for 6 h, 36.8 μl of ddH_2_O was added. The sample was then filtered and analyzed by UPLC-ESI-MS/MS (Waters, Acquity UPLC-Quattro Premier XE MS/MS, Massachusetts, America). 5mC, 5hmC, 5fC, and 5caC standards were mixed in different concentrations and used to generate standard curves.

### Immunohistochemical Staining and Semiquantitative Method

Slides containing 3 μm sections were baked at 60°C for 3 h. The sections were then soaked in xylene I for 15 mins, xylene II for 15 mins, 100% alcohol I for 10 mins, 100% alcohol II for 10 mins, 95% alcohol I for 10 mins, 95% alcohol II for 10 mins, 90% alcohol for 10 mins, 80% alcohol for 5 mins and 70% alcohol for 5 mins. Sildes were washed in phosphate buffer solution (PBS) afterwards. The antigens were recovered using 93–95°C citrate salt for more than 20 mins. Endogenous enzymes were inactivated using 3% H_2_O_2_ for 10 mins and were blocked using 5% sheep serum for 1 h. Rabbit anti-TET2 antibody (1:500, Genetex, GTX124205, Texas, America) was pipetted onto the slides and incubated for 12 h at 4°C. Slides were washed and incubated for 75 mins with horse anti-rabbit antibody (Vector, MP-7401, Beijing, China) at 37°C. After being washed in PBS, the slides were reactivated using a DAB solution. We randomly chose 3–5 fields of view (about 2,000 cells/view slide) for every slide using Pannoramic scanner software. This software can recognize the nucleus and cytoplasm. The cells were separated into strong positive, positive, weak positive and negative groups. H-score=percent of strong positive cells × 100 × 3+ percent of positive cells × 100 × 2+ percent of weak positive cells × 100 × 1- H-score of negative control. Then, we determined TET2 total, nuclear and cytoplasm expression with TET2 total, nuclear, and cytoplasm H-scores.

### PCR and Sequencing

We used primers of *TET2* from other papers (17, 18) and designed primers using the primer design tool in NCBI. Two × Taq PCR StarMix (GenStar, Cat#A112-100-01, Beijing, China) was used.

### Statistical Analysis

IBM SPSS Statistics (20.0 for Mac) and GraphPad Prism (6.0c for Mac) were used to conduct data analysis. *T*-test, Welch's *t-*test, Mann-Whitney U test, chi-squared test and Fisher exact test were used to compare data between 2 groups. ANOVA was used to compare data in more than 3 groups. Pearson and Spearman analyses were used for correlation analysis. Data was shown as the mean ± standard deviation, median (the 25th percentile, the 75th percentile) or percentage. Data analysis was conducted using two-sided test. *P* < 0.05 indicated a significant difference.

### Ethics Approval

Our study received approval from the PUMCH Ethics Committee. The project design was scientific. Subjects' risks and benefits were reasonable.

## Results

### Genomic 5hmC Levels Were High in Normal Pituitary Glands and Lost in NFPAs

**Figure 1:**
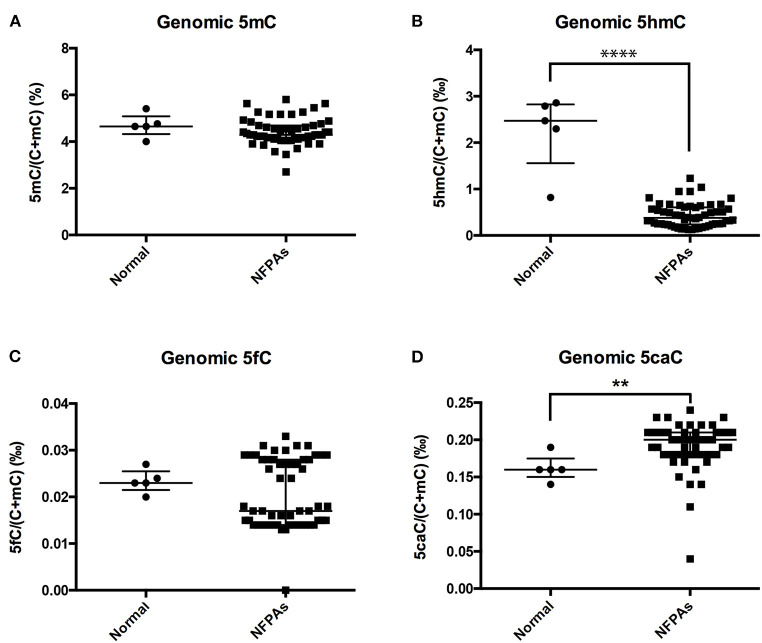
Genomic 5mC, 5hmC, 5fC, and 5caC levels in NFPAs and normal pituitary glands. **(A)** There was no significant difference in genomic 5mC levels between NFPAs and normal pituitary glands. **(B)** Genomic 5hmC levels were significantly decreased in NFPAs compared with those in normal pituitary glands. **(C)** There was no significant difference in genomic 5fC levels between NFPAs and normal pituitary glands. **(D)** Genomic 5caC levels were significantly increased in NFPAs compared with those in normal pituitary glands. **P < *0.01;* ****P < *0.0001*.

Compared with those in normal pituitary glands, genomic 5hmC levels were significantly decreased in NFPAs [0.38‰ (0.24‰, 0.61‰) vs. 2.47‰ (1.56‰, 2.83‰), *P* < 0.0001], and genomic 5caC levels were significantly increased [0.20‰ (0.18‰, 0.21‰) vs. 0.16‰ (0.15‰, 0.18‰), *P* = 0.005] (Figures 1B,D). There was no significant difference in genomic 5mC or 5fC levels in NFPAs and normal pituitary glands [4.29% (4.11%, 4.69%) vs. 4.65% (4.33%, 5.09%), *P* = 0.232, 0.017‰ (0.014‰, 0.028‰) vs. 0.023‰ (0.022‰, 0.025‰), *P* = 0.580] (Figures 1A,C). Data for UPLC-ESI-MS/MS was shown in Supplementary Table 2.

### NFPA Genomic 5hmC Levels Showed No Difference Between Invasive and Non-invasive NFPAs

**Figure 2:**
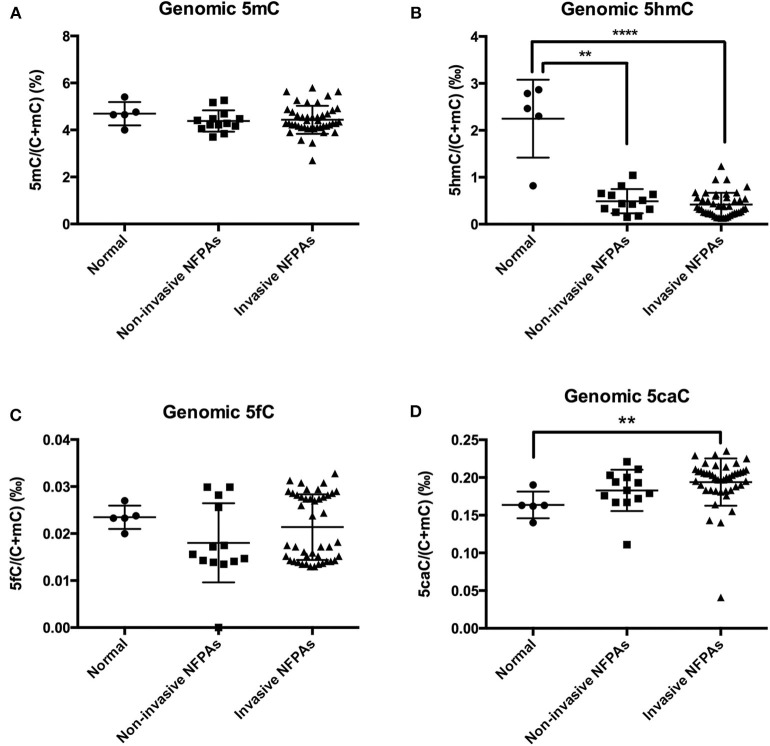
Genomic 5mC, 5hmC, 5fC, and 5caC levels in invasive NFPAs, non-invasive NFPAs and normal pituitary glands. **(A)** There was no significant difference in genomic 5mC among normal pituitary glands, non-invasive NFPAs and invasive NFPAs. **(B)** Genomic 5hmC levels in non-invasive and invasive NFPAs were significantly decreased compared with those in normal pituitary glands. But there was no significant difference in genomic 5hmC levels in non-invasive and invasive NFPAs. **(C)** There was no significant difference in genomic 5fC among normal pituitary glands, non-invasive NFPAs and invasive NFPAs. **(D)** Genomic 5caC levels in invasive NFPAs were significantly increased compared with those in normal pituitary glands. There was no significant difference in genomic 5caC levels in non-invasive NFPAs and normal pituitary glands. There was no significant difference of genomic 5caC levels in non-invasive and invasive NFPAs. **P < *0.01;* ****P < *0.0001*.

Compared with those in normal pituitary glands, genomic 5hmC levels in non-invasive and invasive NFPAs were significantly decreased [0.49 ± 0.26‰ vs. 2.25 ± 0.83‰, *P* = 0.008, 0.35‰ (0.23‰, 0.57‰) vs. 2.47‰ (1.56‰, 2.83‰), *P* < 0.0001] (Figure 2B). However, there was no difference in genomic 5hmC levels in non-invasive and invasive NFPAs [0.44‰ (0.29‰, 0.65‰) vs. 0.35‰ (0.23‰, 0.57‰), *P* = 0.30] (Figure 2B). Genomic 5caC levels in invasive NFPAs were greatly increased compared with those in normal pituitary glands [0.20‰ (0.18‰, 0.21‰) vs. 0.16‰ (0.15‰, 0.18‰), *P* = 0.004] (Figure 2D). There was no significant difference in genomic 5caC levels in non-invasive NFPAs and normal pituitary glands (0.18 ± 0.03‰ vs. 0.16 ± 0.02‰, *P* = 0.168) (Figure 2D). There was no significant difference of genomic 5caC levels in non-invasive and invasive NFPAs [0.18‰ (0.17‰, 0.20‰) vs. 0.20‰ (0.18‰, 0.21‰), *P* = 0.068] (Figure 2D). There was no significant difference in genomic 5mC, 5fC levels among the 3 groups (Figures 2A,C).

### NFPAs With Lower 5hmC Were Larger and Had a Higher Ki-67 Index

When genomic 5hmC levels were more than 0.41‰, the sum of the sensitivity and specificity to identify invasive PAs was largest (Supplementary Figure 1). Thus, 0.41‰ was defined as the cutoff value for genomic 5hmC levels, and samples were separated into 2 groups. Tumors with lower genomic 5hmC levels were larger in diameter (34.6 ± 12.7 mm vs. 27.4 ± 8.5 mm, *P* = 0.023) and had a higher Ki-67 index (9/5 vs. 16/0, *P* = 0.014) (Table 1). Age, gender, course of disease, invasion, P53 and Knosp grade between the 2 groups were not significantly different. The genomic 5hmC levels in samples with a Ki-67 index higher than 3% were decreased significantly (Table 2).

**Table 1:** Relation between 5hmC levels and clinical characteristics in NFPA patients.

	**Sum**	**5hmC<0.41‰**	**5hmC≥0.41‰**	***P*-value**
Age (year)	57	49.9 ± 14.8 (*n* = 31)	52.9 ± 10.0 (*n* = 26)	0.374^a^
Gender	57			0.253^b^
Male	31	19 (61.3%)	12 (38.7%)	
Female	26	12 (46.2%)	14 (53.8%)	
Course of disease (month)	51	12.0 (1.0, 48.0) (*n* = 27)	12.0 (2.5, 51.0) (*n* = 24)	0.977^c^
Invasion	57			0.189^b^
Non-invasive	13	5 (16.1%)	8 (30.8%)	
Invasive	44	26 (83.9%)	18 (69.2%)	
Tumor diameter (mm)	48	34.6 ± 12.7 (*n* = 27)	27.4 ± 8.5 (*n* = 21)	^*^0.023^a^
Ki-67	30			^*^0.014^d^
≤ 3%	25	9 (36.0%)	16 (64.0%)	
>3%	5	5 (100.0%)	0 (0.0%)	
P53	29			0.632^d^
–	24	10 (41.7%)	14 (58.3%)	
+	5	3 (60.0%)	2 (40.0%)	
Knosp grade	12			1.000^d^
0–2 grades	4	2 (50.0%)	2 (50.0%)	
3–4 grades	8	3 (37.5%)	5 (62.5%)	

a Welch's t-test,

b chi-square test,

c Mann-Whitney U test,

d fisher exact test, tumor diameter represented by the largest diameter.

* *P < 0.05*.

**Table 2:** Relation between Ki-67 index and 5mC, 5hmC, 5fC, and 5caC levels.

	**Ki-67 (*****n*** **= 30)**					***P*-value**
	**<1% (*n* = 4)**	**1% (*n* = 9)**	**2% (*n* = 8)**	**3% (*n* = 4)**	**>3% (*n* = 5)**	
5mC(C+mC) (%)	4.51 ± 0.65	4.58 ± 0.67	4.31 ± 0.33	4.44 ± 0.53	4.37 ± 0.31	0.853
5hmC(C+mC) (‰)	0.62 ± 0.16	0.62 ± 0.37	0.45 ± 0.25	0.47 ± 0.19	0.15 ± 0.02	^*^0.040
5fC(C+mC)	0.014	0.018	0.020	0.022	0.020	0.595
(‰)	± 0.010	± 0.005	± 0.008	± 0.007	± 0.008	
5caC(C+mC) (‰)	0.17 ± 0.04	0.18 ± 0.03	0.20 ± 0.02	0.21 ± 0.02	0.20 ± 0.02	0.081

ANOVA,

* *P < 0.05*.

### TET2 Localized in the Nucleus and Cytoplasm of the Normal Pituitary Gland and NFPAs

**Figure 3:**
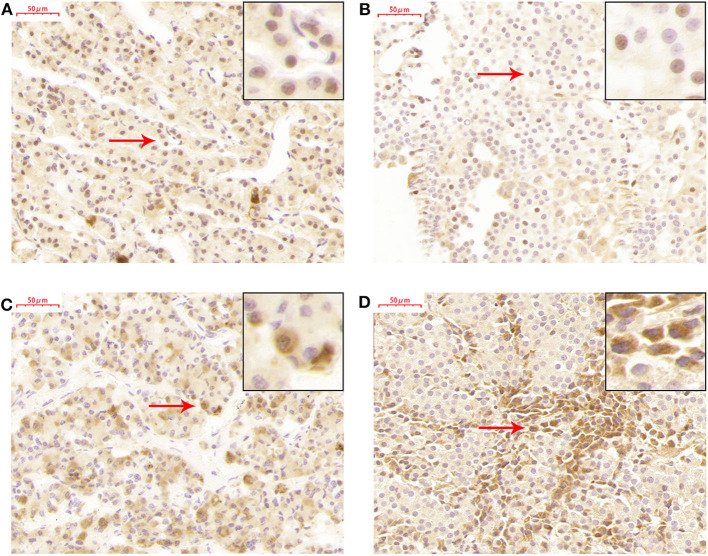
TET2 expression and localization in the normal pituitary gland and NFPAs. **(A)** TET2 localized in the nucleus of the normal pituitary gland. **(B)** TET2 also localized in the nucleus of NFPAs. **(C)** TET2 localized in the cytoplasm of the normal pituitary gland. **(D)** TET2 also localized in the cytoplasm of NFPAs.

TET2 immunohistochemistry was performed in a normal pituitary and 26 NFPAs. The results revealed that TET2 localized in both the nucleus and cytoplasm of the normal pituitary gland and NFPAs (Figure 3). The expression and localization of TET2 were different in different kinds of cells (Figure 3). Genomic 5hmC levels between non-invasive and invasive NFPAs were not significantly different (Supplementary Figure 2). TET2 total, nuclear and cytoplasmic expression in non-invasive and invasive NFPAs was also not significantly different (Supplementary Figure 2). All immunohistochemical staining data was provided in Supplementary Table 2.

### NFPA Genomic 5hmC Levels Were Related With TET2 Total Expression and Nuclear Localization

**Figure 4:**
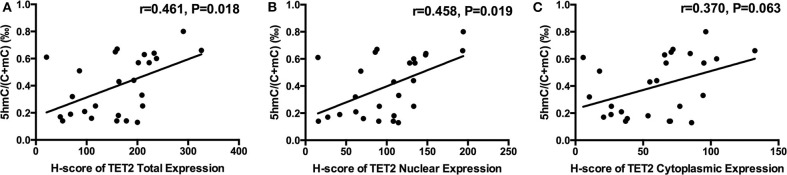
Relation between NFPA genomic 5hmC and TET2 expression and localization. **(A)** NFPA genomic 5hmC levels were positively related with TET2 total expression. **(B)** NFPA genomic 5hmC levels were also positively related with TET2 nuclear expression. **(C)** NFPA genomic 5hmC levels were not significantly related with TET2 cytoplasmic expression.

There was a trend toward an increase in genomic 5hmC levels in NFPAs along with an increase in TET2 total, nuclear and cytoplasmic expression (Figure 4). NFPA genomic 5hmC levels were positively correlated with TET2 total and nuclear expression (*r* = 0.461, *P* = 0.018, *r* = 0.458, *P* = 0.019) but not correlated with TET2 cytoplasmic expression (*r* = 0.370, *P* = 0.063) (Figure 4).

### NFPA TET2 Total Expression and Nuclear Localization Were Decreased in the Presence of Low 5hmC Levels

**Figure 5:**
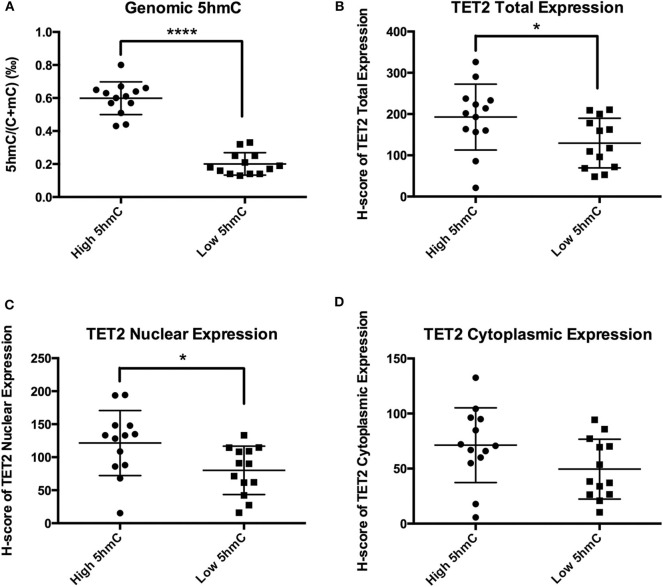
TET2 expression and localization in high and low 5hmC groups. *P < *0.05;* ****P < *0.0001*. **(A)** There were significantly different of 5hmC levels between the low 5hmC group and the high 5hmC group. **(B)** TET2 total expression in the low 5hmC group was lower than that in the high 5hmC group. **(C)** TET2 nuclear expression in the low 5hmC group was lower than that in the high 5hmC group. **(D)** There was no significant difference in TET2 cytoplasmic localization between the low 5hmC group and the high 5hmC group.

TET2 immunohistochemistry samples were separated into 2 groups at the 5hmC level of 0.41‰. 5hmC levels in the 2 groups were significantly different (0.60 ± 0.10‰ vs. 0.20 ± 0.07‰, *P* < 0.0001) (Figure 5A). TET2 total expression and nuclear localization in the low 5hmC group were lower than those in the high 5hmC group (192.78 ± 79.87 vs. 129.58 ± 60.18, *P* = 0.032, 121.49 ± 49.21 vs. 80.07 ± 36.68, *P* = 0.023) (Figures 5B,C). There was no significant difference in TET2 cytoplasmic localization between the 2 groups (Figure 5D).

### Genomic 5hmC Levels of NFPAs With TET2 p.P29R Were Lower Than Those in Wild Type

We chose 6 NFPAs with low 5hmC levels to sequence *TET2* exons 3–11 (exons 1 and 2 are non-coding regions). Primers for the *TET2* coding regions were shown in Supplementary Table 1. We found 3 single nucleotide polymorphism (SNP) sites in 5 NFPAs: *TET2* c. 86C>G, c.5162T>G, and c.5284A>G (plus strand), leading to TET2 p.P29R, p.L1721W, and p.I1762V (Supplementary Figure 3). Then, we sequenced another 51 NFPAs with primers for 3 SNP sites.

**Figure 6:**
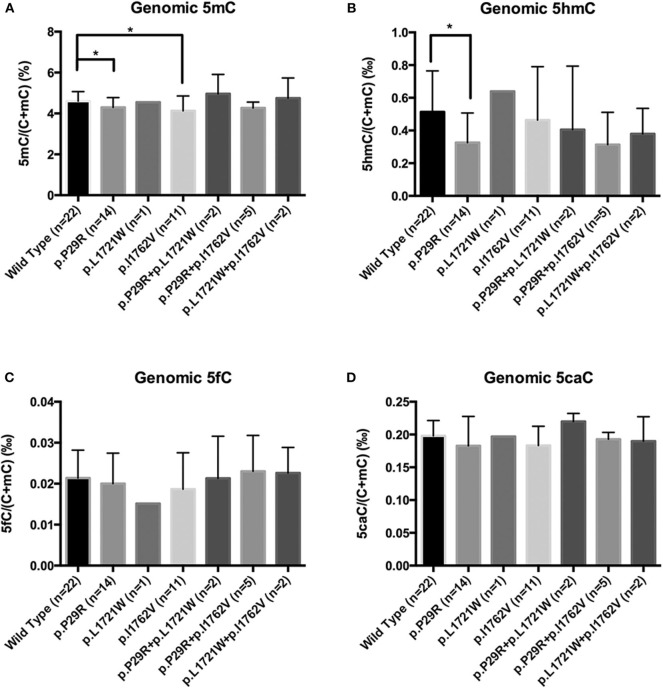
Genomic 5mC, 5hmC, 5fC, and 5caC levels in NFPAs with various SNP sites. *P < *0.05*. **(A)** Genomic 5mC levels in the TET2 p.P29R and TET2 p.I1762V groups were lower than that in the wild-type group. **(B)** Genomic 5hmC level in the TET2 p.P29R group was lower than that in the wild-type group. **(C)** There was no significant difference of 5fC among the 7 groups. **(D)** There was no significant difference of 5caC among the 7 groups.

There were few homozygous variations, and genomic 5hmC levels in homozygous variation samples were similar to heterozygous variation samples in the same site. Thus, we merged heterozygous variation NFPAs and homozygous variation NFPAs in the same site into a single group. Samples were separated into 7 groups. NFPAs in the wild-type group had the same sequence as the NCBI reference sequence. The results showed that the TET2 p.P29R group had lower genomic 5hmC levels (0.33 ± 0.18‰ vs. 0.51 ± 0.25‰, *P* = 0.021) while the TET2 p.P29R and TET2 p.I1762V groups had lower genomic 5mC levels than those in the wild-type group [4.17% (4.05%, 4.52%) vs. 4.48% (4.28%, 4.89%), *P* = 0.027, 4.13 ± 0.73% vs. 4.60 ± 0.46%, *P* = 0.029] (Figure 6). 5fC and 5caC showed no significant difference among the 7 groups (Figure 6).

### NFPA TET2 p.P29R Did Not Influence TET2 Expression and Localization

**Figure 7:**
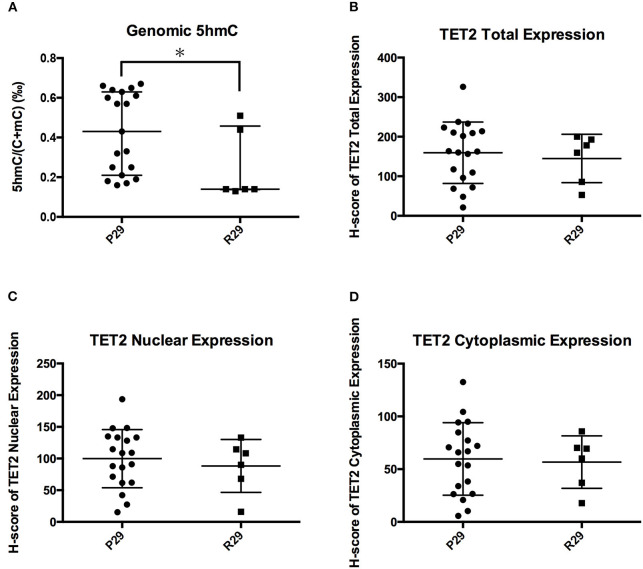
Influence of TET2 p.P29R on TET2 expression and localization. **(A)** Genomic 5hmC levels in the R29 group were lower than those in the P29 group. **(B)** TET2 total expression was not significantly different between the R29 group and the P29 group. **(C)** TET2 nuclear localization was not significantly different between the R29 group and the P29 group. **(D)** TET2 cytoplasmic localization was not significantly different between the R29 group and the P29 group. *P < *0.05*.

Figure 6 showed that the TET2 p.P29R group had lower genomic 5hmC levels compared with the wild-type group. We separated 26 NFPAs that were analyzed with TET2 immunohistochemistry into 2 groups according to TET2 p.P29R variation. There was only one exceptional sample, the 5hmC level in which made no difference between the R29 and P29 groups (NFPAs without TET2 p.P29R were defined as P29 group. NFPAs with TET2 p.P29R were defined as R29 group). So, we excluded a sample with exceptional 5hmC level and included 25 NFPAs. The results showed that genomic 5hmC levels in the R29 group were lower than those in the P29 group [0.140‰ (0.138‰, 0.458‰) vs. 0.430‰ (0.210‰, 0.630‰), *P* = 0.017] (Figure 7A), but TET2 total expression and nuclear and cytoplasmic localization were not significantly different between the 2 groups (144.95 ± 61.06 vs. 159.50 ± 77.81, *P* = 0.680, 88.26 ± 41.79 vs. 99.82 ± 45.90, *P* = 0.589, 56.68 ± 24.88 vs. 59.69 ± 34.40, *P* = 0.846) (Figure 7).

## Discussion

With the development of epigenetic studies, more and more researchers have found that changes in epigenetic modification are associated with tumorigenesis. Genomic 5hmC levels in tumors are significantly different from those in normal tissue. Additionally, methylation changes in DNA have been revealed in PAs. However, genomic 5hmC levels in PAs have not been reported. Our study chose normal pituitary glands as control. Normal pituitary glands were obtained from corpses dying from liver cancer, breast cancer, diabetes mellitus, pharyngeal cancer, intestinal cancer, and heart disease, without clinical manifestations of cerebral disease. We investigated genomic 5hmC levels in NFPAs and the underlying mechanism to determine the role of epigenetics in PAs.

In our study, genomic 5hmC levels in NFPAs largely decreased to 1/5 of those in normal pituitary glands. This phenomenon implies that changes in hydroxymethylation may play a role in NFPA tumorigenesis. Genomic 5mC levels showed no significant difference between NFPAs and normal pituitary glands. As a precursor of 5hmC, the amount of 5mC may change along with 5hmC levels. However, genomic 5mC levels are 20–100 times of genomic 5hmC levels. This may explain why there was no significant difference in genomic 5mC levels between NFPAs and normal pituitary glands even though genomic 5hmC levels decreased. Kober *et al*. reported the significant increase of genome-wide DNA methylation in human NFPAs compared with normal pituitary glands (6). This discrepancy may be due to limited samples and different detection methods. Genomic 5caC levels in NFPAs were higher than those in normal pituitary glands because 5caC is an end-product of 5mC under TET catalyzation. So, the increase in 5caC may reveal changes in demethylation activity, but further studies are needed to explore the mechanism that generates high 5caC levels in NFPAs. 5fC is a temporary intermediate product of 5mC under TET catalyzation and amounts to 1/2000 of 5mC. Therefore, genomic 5fC levels in NFPAs were not significantly different from those of normal pituitary glands.

Many studies have shown a relation between genomic 5hmC levels and tumor invasion. Decrease in genomic 5hmC levels can indicate increase in tumor invasion and is considered a poor prognosis factor in glioma (19), breast cancer (11) and melanoma (10). However, genomic 5hmC levels in invasive NFPAs were not significantly different from those in non-invasive NFPAs in our study. The different results may be due to different types of tumors since glioma, breast cancer and melanoma are malignant tumors, while NFPAs are benign tumors. Although there was no difference in genomic 5hmC levels, 5hmC distribution in a single gene, especially a tumor-related gene, may be different between invasive NFPAs and non-invasive NFPAs. Exploration of 5hmC distribution in a single gene between invasive and non-invasive NFPAs may reveal information about the mechanism of NFPA invasion.

We analyzed the relationship between genomic 5hmC levels and clinical information. NFPAs with lower 5hmC had a larger diameter and higher Ki-67 index. 5hmC levels in NFPAs with a high Ki-67 index (>3%) were 1/4–1/3 of those in other NFPAs. Lian et al. (10) found that tumors with low 5hmC levels were larger than those with high 5hmC levels 10 days after inoculation in nude mice. Therefore, lower 5hmC levels may indicate faster proliferation and larger tumor volume (10).

We investigated genomic 5mC, 5hmC, 5fC, and 5caC levels in NFPAs. Genomic cytosine modifications are the sum of cytosine modifications to all genes. Further studies should be conducted regarding 5mC, 5hmC, 5fC, and 5caC in single genes. Changes in 5hmC in a single gene can change the expression of this gene. Bhattacharyya et al. (5) found that 5hmC in the promoter region of the oncogene *GATA6* increased along with the decrease of 5mC, and *GATA6* expression increased in pancreatic cancer cells compared with normal cells. The genomic 5hmC levels in NFPAs were significantly different, so there must be more changes in 5hmC in many genes.

The TET family plays its role in the nucleus, but studies have reported TET family members in the cytoplasm. Stefano et al. (20) found that loss of C/EBPα resulted in TET2 in the cytoplasm of B cells. Müller et al. (21) found TET1 in both the nucleus and cytoplasm in gliomas. Localization of the TET family in the cytoplasm is common in tumor tissues and related with tumor invasion. Huang et al. (16) found that TET2 and TET3 were mainly in the nucleus in normal colon tissue but that TET2 was mainly in the cytoplasm in colon cancer tissue, especially in distal metastasis tissue. However, TET2 was located in both the nucleus and cytoplasm in NFPAs and the normal pituitary in our study. In order to maintain original cell feature, only fresh tissue can be used for immunohistochemical staining. It is really difficult to get fresh normal pituitary glands. So, we used only one normal pituitary for TET2 immunohistochemistry. More samples should be used to confirm this result.

There is an exact relationship between genomic 5hmC levels and TET expression and localization. Overexpression of TET2 in melanoma cells corresponds to higher 5hmC levels (10). Cytoplasmic localization of TET1 corresponds to loss of 5hmC in gliomas (21). In our study, genomic 5hmC levels were positively correlated to TET2 total and nuclear expression in NFPAs. TET2 total and nuclear expression in the high 5hmC group was significantly greater than that in the low 5hmC group. There is a close relationship between 5hmC levels and TET2 expression and subcellular localization.

*TET2* mutations are common in myeloid diseases and are directly related with 5hmC levels (14, 15, 22, 23). Additionally, there are *TET2* mutations in breast cancer (24). Breast cancer samples with *TET2* mutations showed lower 5hmC levels than those of samples without *TET2* mutations (24). However, we did not find a *TET2* mutation in 6 NFPA sequences of all coding regions. *TET2* mutations may be not common in NFPAs. *TET2* is located in 4q24, where the ectopic breaking point of myeloid disease is located. For example, acute myelocytic leukemia is related to t(3;4)(q26;q24), t(4;5)(q24;p16), and t(4;7)(q24;q21) (23, 25). Therefore, *TET2* mutations are common in myeloid diseases. However, we only sequenced all coding regions in 6 samples. These results were limited by sample size.

Among 3 *TET2* SNP sites, only TET2 p.P29R variation was related with reduced 5hmC levels. However, TET2 total expression and nuclear and cytoplasmic localization were not different between the P29 and R29 groups. One reason is that the results were limited by the quantity of immunohistochemistry samples. Another reason is that TET2 p.P29R may influence TET2 function. However, the twenty-ninth amino acid of TET2 is located in a non-catalyzing region; therefore, it may not affect TET2 catalytic function. There is no study regarding the functional verification of TET2 p.P29R variation (26, 27). Chou et al. (28) reported that TET2 p.P29R did not affect survival time. Furthermore, TET2 interacts with IDAX through its C-terminus (12). So TET2 p.P29R may not influence an interaction with IDAX or identification of DNA. Overall, there are not enough evidences to prove the effect of TET2 p.P29R on TET2 expression, localization and function. The relation between TET2 p.P29R and low 5hmC can not be well-explained. However, the TET2 catalytic reaction is a complex process associated with many proteins and factors. Whether TET2 p.P29R influences other proteins and factors in this reaction is unknown.

## Conclusions

We studied genomic 5hmC levels in NFPAs and explored the mechanism of 5hmC changes. We think that changes in DNA hydroxymethylation and 5hmC distribution patterns may play important roles in the formation of NFPAs. Although genomic 5hmC levels between invasive and non-invasive NFPAs were not significantly different, changes in 5hmC distribution patterns may exist in many genes. Additionally, lower 5hmC levels may indicate stronger NFPA proliferation and larger tumor volume. As to the mechanism of 5hmC changes, we think that *TET2* mutations may be not common in NFPAs and can not explain 5hmC loss. TET2 p.P29R was related with low 5hmC levels. However, we do not know how this affects protein function. Genomic 5hmC levels were closely related with TET2 total expression and nuclear localization. So TET2 expression and subcellular localization may affect 5hmC levels.

## Data Availability Statement

The datasets analyzed for this study can be found in the NCBI (http://www.ncbi.nlm.nih.gov/bioproject/630023). The accession number is PRJNA630023.

## Ethics Statement

The studies involving human participants were reviewed and approved by Ethics Committee of PUMCH. The patients/participants provided their written informed consent to participate in this study.

## Author Contributions

YX conducted all experiments. YN and W-MT helped to complete the UPLC-ESI-MS/MS experiment. KD and RW helped to separate NFPAs into invasive and non-invasive groups. HP and FG helped to conduct immunohistochemical experiment. FF and HY provided and analyzed NFPAs MRI. SC and LL helped with PCR and sequencing experiment. YY and HZ designed all the experiments.

## Conflict of Interest

The authors declare that the research was conducted in the absence of any commercial or financial relationships that could be construed as a potential conflict of interest.

## Supplementary Material

Supplementary Figure 1 Receiver operating characteristic (ROC) curves of 5hmC **(A)** and 5caC **(B)** to identify NFPA invasion. Genomic 5hmC and 5caC levels in NFPAs were significantly different from those in normal pituitary glands. This revealed that 5hmC and 5caC might play important roles in the epigenetic modification of NFPAs. So, we used genomic 5hmC and 5caC to identify NFPA invasion by ROC curve analysis. The areas of ROC curves were 0.595 ± 0.088 (*P* = 0.300) and 0.668 ± 0.087 (*P* = 0.068). Genomic 5hmC or 5caC levels cannot be used to identify NFPA invasion.

Supplementary Figure 2 TET2 expression and localization in invasive and non-invasive NFPAs. A total of 26 NFPAs analyzed by TET2 immunohistochemistry were separated into invasive and non-invasive groups. **(A)** Genomic 5hmC levels between the 2 groups were not significantly different [0.44‰ (0.30‰, 0.62‰) vs. 0.23‰ (0.15‰, 0.63‰), *P* = 0.363]. **(B-D)** TET2 total, nuclear and cytoplasmic expression in the 2 groups was also not significantly different (165.74 ± 87.44 vs. 158.33 ± 71.57, *P* = 0.815, 108.52 ± 50.06 vs. 95.95 ± 46.78, *P* = 0.522, 57.22 ± 39.39 vs. 62.39 ± 27.87, *P* = 0.698).

Supplementary Figure 3 *TET2* variations and amino acid sites in the protein. There were 3 SNP variations in nucleotide sequences and amino acid sequences. **(A–C)** Showed wild type. **(D–F)** Showed SNP sites. ^*^Represented variation sites. **(G)** TET2 p.P29R is located in the N-terminus. TET2 p.L1721W and TET2 p.I1762V are located in a non-conserved region of double-stranded β-helix (DSBH). A portion of the picture is from Mohr et al. (14).

Supplementary Table 1 Primers for *TET2* coding region.

Supplementary Table 2 Data for UPLC-ESI-MS/MS and immunohistochemical staining.
