# Supplementary figures and images for: Changes in DNA 5-Hydroxymethylcytosine Levels and the Underlying Mechanism in Non-functioning Pituitary Adenomas
Source: Front Endocrinol (Lausanne). 2020 Jul 8;11:361. doi: 10.3389/fendo.2020.00361 (PMC7381329; doi:10.3389/fendo.2020.00361)

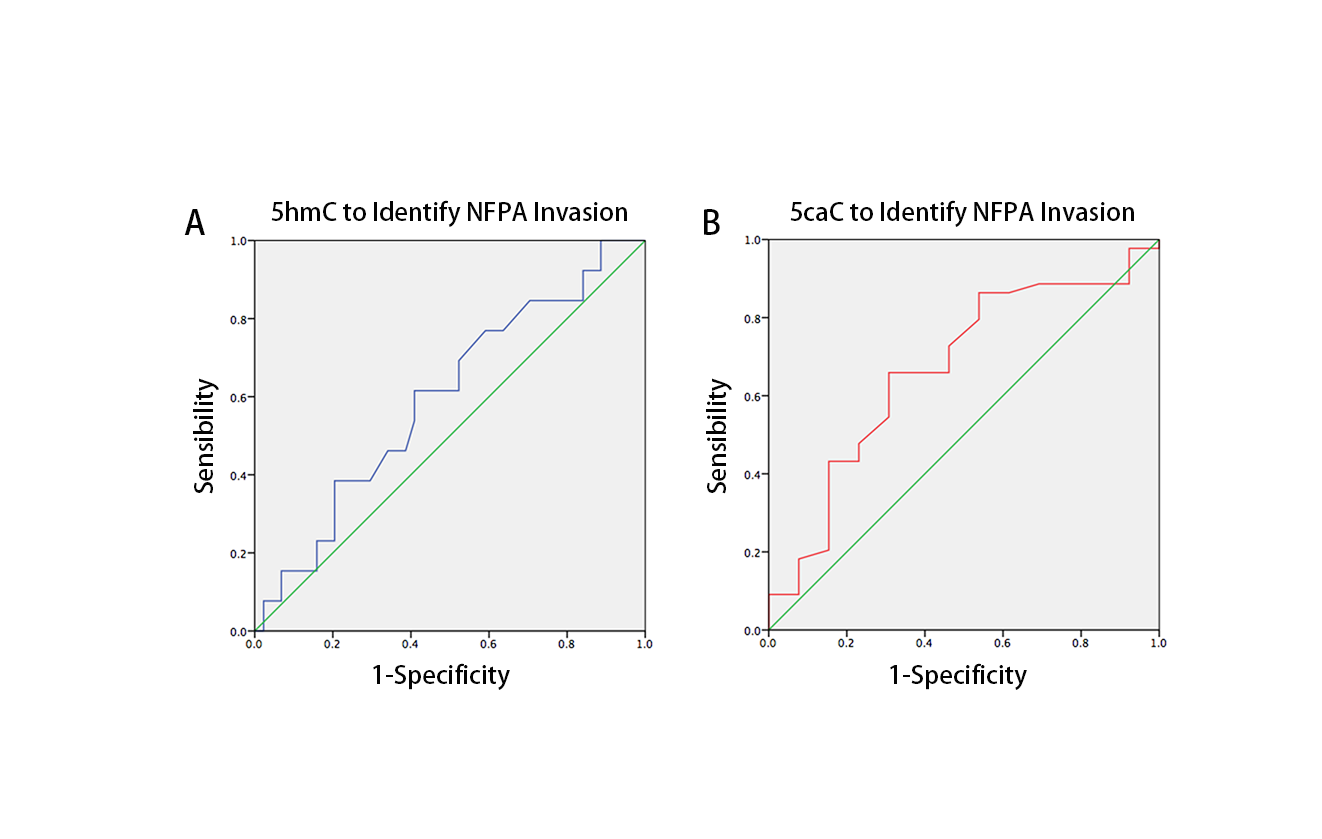

Supplement: Supplementary Figure 1 — Receiver operating characteristic (ROC) curves of 5hmC (A) and 5caC (B) to identify NFPA invasion. Genomic 5hmC and 5caC levels in NFPAs were significantly different from those in normal pituitary glands. This revealed that 5hmC and 5caC might play important roles in the epigenetic modification of NFPAs. So, we used genomic 5hmC and 5caC to identify NFPA invasion by ROC curve analysis. The areas of ROC curves were 0.595 ± 0.088 (P = 0.300) and 0.668 ± 0.087 (P = 0.068). Genomic 5hmC or 5caC levels cannot be used to identify NFPA invasion. [file Image_1.tif]

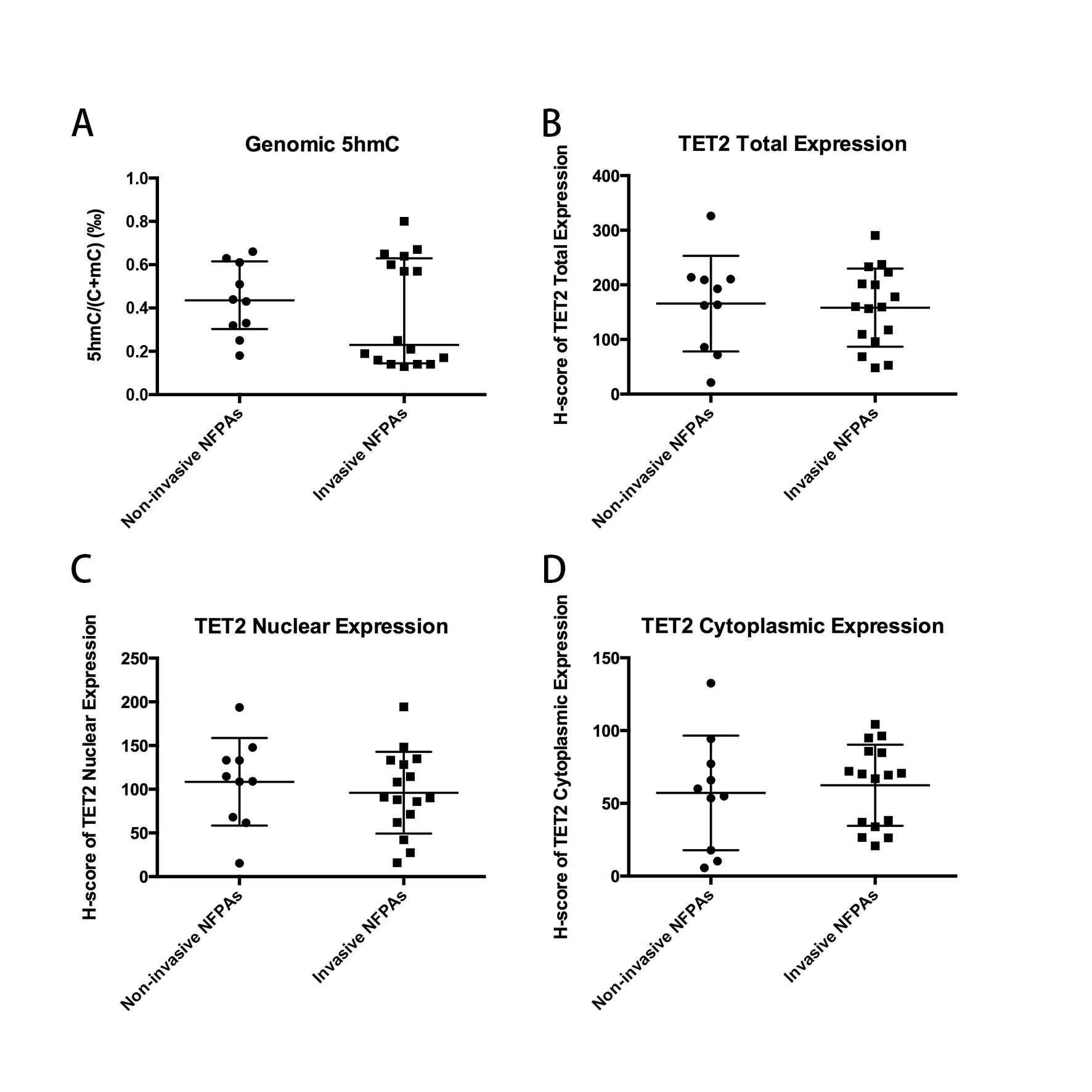

Supplement: Supplementary Figure 2 — TET2 expression and localization in invasive and non-invasive NFPAs. A total of 26 NFPAs analyzed by TET2 immunohistochemistry were separated into invasive and non-invasive groups. (A) Genomic 5hmC levels between the 2 groups were not significantly different [0.44‰ (0.30‰, 0.62‰) vs. 0.23‰ (0.15‰, 0.63‰), P = 0.363]. (B-D) TET2 total, nuclear and cytoplasmic expression in the 2 groups was also not significantly different (165.74 ± 87.44 vs. 158.33 ± 71.57, P = 0.815, 108.52 ± 50.06 vs. 95.95 ± 46.78, P = 0.522, 57.22 ± 39.39 vs. 62.39 ± 27.87, P = 0.698). [file Image_2.tif]

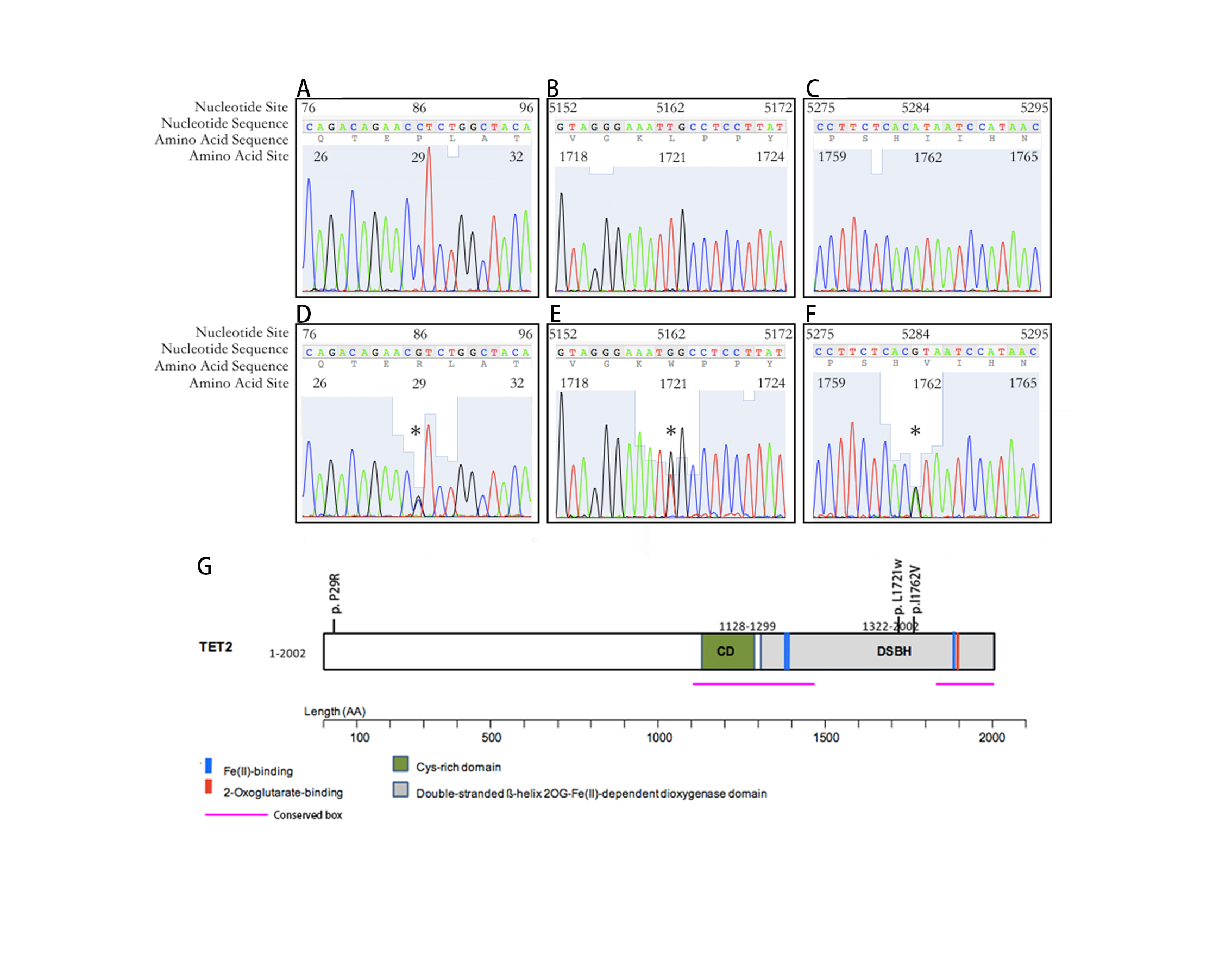

Supplement: Supplementary Figure 3 — TET2 variations and amino acid sites in the protein. There were 3 SNP variations in nucleotide sequences and amino acid sequences. (A–C) Showed wild type. (D–F) Showed SNP sites. *Represented variation sites. (G) TET2 p.P29R is located in the N-terminus. TET2 p.L1721W and TET2 p.I1762V are located in a non-conserved region of double-stranded β-helix (DSBH). A portion of the picture is from Mohr et al. (14). [file Image_3.TIF]
