# Supplementary material for: Changes in DNA 5-Hydroxymethylcytosine Levels and the Underlying Mechanism in Non-functioning Pituitary Adenomas
Source: Front Endocrinol (Lausanne). 2020 Jul 8;11:361. doi: 10.3389/fendo.2020.00361 (PMC7381329; doi:10.3389/fendo.2020.00361)
Supplement: Supplementary Table 1 — Primers for TET2 coding region. [file Data_Sheet_1.docx]

**SUPPLEMENTARY INFORMATION**

## Primers for *TET2* PCR and sequencing

**SUPPLEMENTARY TABLE 1 |** Primers for *TET2* coding region

| Order | Name | Orientation | Sequence | Length (bp) |
| --- | --- | --- | --- | --- |
| 1 | 620 | F | TGAACTTCCCACATTAGCTGGT | 955 |
|  |  | R | GAAACTGTAGCACCATTAGGCATT |  |
| 2 | 596 | F | CAAAAGGCTAATGGAGAAAGACGTA | 836 |
|  |  | R | GCAGAAAAGGAATCCTTAGTGAACA |  |
| 3 | 578 | F | ACTCACCCATCGCATACCTC | 887 |
|  |  | R | AGATAGTGCTGTGTTGGGGG |  |
| 4 | 579 | F | TTCCACAGGTTCCTCAGCTT | 754 |
|  |  | R | GAGAAGTGCACCTGGTGTGA |  |
| 5 | 621 | F | GACCAATGTCAGAACACCTCAA | 867 |
|  |  | R | TTGATTTTGAATACTGATTTTCACCA |  |
| 6 | 622 | F | TTGCAACATAAGCCTCATAAACAG | 788 |
|  |  | R | ATTGGCCTGTGCATCTGACTAT |  |
| 7 | 581 | F | AATGTCCAAATGGGACTGGA | 785 |
|  |  | R | ACTGGCCCTGACATTTCAAC |  |
| 8 | 582 | F | CCCCAGAAGGACACTCAAAA | 698 |
|  |  | R | CAAATTGCTGCCAGACTCAA |  |
| 9 | 583 | F | ACTTGATAGCCACACCCCAG | 685 |
|  |  | R | TTCCCCCAACTCATGAAGAC |  |
| 10 | 619 | F | AGCCCTTAATGTGTAGTTGGGG | 344 |
|  |  | R | GCACAGTGTGTAGTGTTGGC |  |
| 11 | 623 | F | CATTTCTCAGGATGTGGTCATAGAAT | 286 |
|  |  | R | CCCAATTCTCAGGGTCAGATTTA |  |
| 12 | 586 | F | AGACTTTATGTATCTTTCATCTAGCTCTGG | 595 |
|  |  | R | ACTCTCTTCCTTTCAACCAAAGATT |  |
| 13 | 587 | F | GTGTGGTTATGCCACAGCTT | 501 |
|  |  | R | CCAAAGAGGAAGTTTTTGTTGC |  |
| 14 | 588 | F | ACCATACGGCTTAATTCCCC | 422 |
|  |  | R | TGTTACAATTGCTGCCAATGA |  |
| 15 | 597 | F | TGTCATTCCATTTTGTTTCTGGATA | 361 |
|  |  | R | AAATTACCCAGTCTTGCATATGTCTT |  |
| 16 | 598 | F | CTGGATCAACTAGGCCACCAAC | 774 |
|  |  | R | CCAAAATTAACAATGTTCATTTTACAATAAGAG |  |
| 17 | 624 | F | GCTCTTATCTTTGCTTAATGGGTGT | 748 |
|  |  | R | TGTACATTTGGTCTAATGGTACAACTG |  |
| 18 | 600 | F | AATGGAAACCTATCAGTGGACAAC | 1107 |
|  |  | R | TATATATCTGTTGTAAGGCCCTGTGA |  |
| 19 | 594 | F | ATTGGCACTAGTCCAGGGTG | 621 |
|  |  | R | ACTGTGACCTTTCCCCACTG |  |

*F: Forward primer, R: Reverse primer.*

## Data for ultrahigh performance liquid chromatography-electrospray ionization-tandem mass spectrometry (UPLC-ESI-MS/MS) and immunohistochemical staining

## SUPPLEMENTARY TABLE 2 | Data for UPLC-ESI-MS/MS and immunohistochemical staining

| **Number** | **5mC/**  **(C+mC) (%)** | **5hmC/**  **(C+mC) (‰)** | **5fC/**  **(C+mC) (‰)** | **5caC/**  **(C+mC) (‰)** | **TET2** nuclear expression (**H-score)** | **TET2** cytoplasmic expression **(H-score)** | **TET2** total expression (**H-score)** |
| --- | --- | --- | --- | --- | --- | --- | --- |
| A1 | 3.45 | 0.17 | 0.017 | 0.19 | 27.45 | 20.75 | 48.19 |
| A2 | 5.17 | 0.66 | 0.017 | 0.17 | 193.61 | 132.56 | 326.17 |
| A3 | 5.17 | 0.57 | 0.017 | 0.19 | 128.25 | 94.88 | 223.12 |
| A4 | 5.17 | 0.67 | 0.017 | 0.21 | 88.00 | 72.03 | 160.03 |
| A5 | 4.62 | 0.54 | 0.031 | 0.20 |  |  |  |
| A6 | 4.88 | 0.65 | 0.024 | 0.20 | 85.94 | 70.71 | 156.65 |
| A7 | 4.11 | 0.14 | 0.027 | 0.21 | 15.85 | 37.03 | 52.88 |
| A8 | 4.29 | 0.13 | 0.029 | 0.23 | 114.40 | 85.75 | 200.15 |
| A9 | 4.29 | 0.43 | 0.014 | 0.20 | 108.74 | 54.93 | 163.66 |
| A10 | 4.84 | 0.95 | 0.016 | 0.21 |  |  |  |
| A11 | 4.05 | 1.04 | 0.014 | 0.18 |  |  |  |
| A12 | 5.45 | 0.49 | 0.018 | 0.16 |  |  |  |
| A13 | 4.23 | 0.63 | 0.028 | 0.21 | 147.85 | 65.89 | 213.74 |
| A14 | 4.29 | 0.21 | 0.014 | 0.21 |  |  |  |
| A15 | 3.90 | 0.14 | 0.013 | 0.18 | 108.14 | 70.13 | 178.27 |
| A16 | 3.90 | 0.49 | 0.026 | 0.19 |  |  |  |
| A17 | 4.92 | 0.95 | 0.033 | 0.23 |  |  |  |
| A18 | 5.26 | 0.32 | 0.018 | 0.14 |  |  |  |
| A19 | 4.41 | 0.18 | 0.015 | 0.22 | 109.02 | 53.61 | 162.62 |
| A20 | 4.11 | 0.51 | 0.027 | 0.22 |  |  |  |
| A21 | 3.70 | 0.81 | 0.000 | 0.11 |  |  |  |
| A22 | 4.17 | 0.32 | 0.028 | 0.21 |  |  |  |
| A23 | 4.29 | 0.39 | 0.014 | 0.14 |  |  |  |
| A24 | 4.76 | 0.16 | 0.016 | 0.21 | 71.31 | 38.33 | 109.64 |
| A25 | 4.05 | 0.27 | 0.027 | 0.22 |  |  |  |
| A26 | 4.23 | 0.25 | 0.014 | 0.18 | 133.29 | 77.15 | 210.44 |
| A27 | 4.69 | 0.44 | 0.016 | 0.20 | 132.96 | 60.01 | 192.97 |
| A28 | 4.48 | 0.15 | 0.030 | 0.18 |  |  |  |
| A29 | 4.23 | 0.24 | 0.014 | 0.18 |  |  |  |
| A30 | 3.90 | 0.23 | 0.013 | 0.21 |  |  |  |
| A31 | 5.26 | 0.61 | 0.018 | 0.19 | 15.40 | 5.67 | 21.07 |
| A32 | 4.23 | 0.38 | 0.028 | 0.23 |  |  |  |
| A33 | 4.17 | 0.32 | 0.014 | 0.17 | 61.50 | 10.29 | 71.79 |
| A34 | 4.55 | 0.67 | 0.015 | 0.18 |  |  |  |
| A35 | 4.69 | 1.23 | 0.031 | 0.22 |  |  |  |
| A36 | 4.05 | 0.36 | 0.014 | 0.18 |  |  |  |
| A37 | 4.41 | 0.51 | 0.029 | 0.24 |  |  |  |
| A38 | 3.85 | 0.33 | 0.026 | 0.17 | 114.79 | 94.30 | 209.09 |
| A39 | 4.29 | 0.39 | 0.029 | 0.20 |  |  |  |
| A40 | 2.70 | 0.19 | 0.014 | 0.20 | 42.11 | 26.33 | 68.44 |
| A41 | 4.48 | 0.51 | 0.030 | 0.19 | 68.08 | 17.80 | 85.88 |
| A42 | 4.11 | 0.44 | 0.014 | 0.21 |  |  |  |
| A43 | 4.11 | 0.25 | 0.027 | 0.21 | 90.87 | 26.55 | 117.43 |
| A44 | 5.63 | 0.13 | 0.014 | 0.18 |  |  |  |
| A45 | 4.23 | 0.21 | 0.028 | 0.18 | 62.05 | 33.99 | 96.04 |
| A46 | 4.55 | 0.26 | 0.015 | 0.20 |  |  |  |
| A47 | 4.55 | 0.24 | 0.015 | 0.20 |  |  |  |
| A48 | 4.55 | 0.64 | 0.015 | 0.20 | 148.29 | 84.78 | 233.06 |
| A49 | 4.17 | 0.14 | 0.014 | 0.18 | 90.15 | 69.38 | 159.53 |
| A50 | 4.17 | 0.25 | 0.028 | 0.21 |  |  |  |
| A51 | 4.35 | 0.80 | 0.029 | 0.19 | 194.25 | 96.26 | 290.50 |
| A52 | 4.05 | 0.34 | 0.014 | 0.04 |  |  |  |
| A53 | 5.80 | 0.57 | 0.029 | 0.20 | 134.82 | 66.96 | 201.78 |
| A54 | 5.63 | 0.68 | 0.014 | 0.21 |  |  |  |
| A55 | 4.41 | 0.60 | 0.029 | 0.21 | 133.24 | 104.33 | 237.57 |
| A56 | 3.57 | 0.32 | 0.024 | 0.15 |  |  |  |
| A57 | 4.62 | 0.25 | 0.031 | 0.20 |  |  |  |
| N1 | 4.65 | 2.47 | 0.023 | 0.16 |  |  |  |
| N2 | 4.65 | 2.30 | 0.023 | 0.16 |  |  |  |
| N3 | 5.41 | 2.86 | 0.027 | 0.16 |  |  |  |
| N4 | 4.00 | 0.82 | 0.020 | 0.14 |  |  |  |
| N5 | 4.76 | 2.79 | 0.024 | 0.19 |  |  |  |

*N in the number column represents a normal pituitary gland. A in the number column represents* *the nonfunctioning pituitary adenoma. Blank means no experiment.*
